# YOLOv13-SwinTongue: Tongue Coating Diagnosis Using an Enhanced YOLOv13 with Swin Transformer

**DOI:** 10.3390/s26010219

**Published:** 2025-12-29

**Authors:** Xiangqiang Yang, Jinchao Hao, Yonggang Wang, Yunfeng Man, Renjie Yang, Qinge Wu

**Affiliations:** 1 Henan Communications Engineering Co., Ltd., Zhengzhou 450044, China; 2 Environmental Science, The University of Queensland, Brisbane, QLD 4067, Australia; 3 School of Computer and Information Engineering, Shanghai Polytechnic University, Shanghai 201209, China

**Keywords:** tongue coating diagnosis, YOLOv13, swin transformer

## Abstract

Tongue coating is a crucial diagnostic indicator in traditional Chinese medicine, intuitively reflecting the body’s physiological and pathological conditions. However, traditional visual inspection methods are highly susceptible to subjective bias, often resulting in diagnostic deviations and inconsistencies. To address these limitations, this study proposes an intelligent tongue coating diagnostic model based on an enhanced YOLOv13. The model integrates a hybrid architecture of swin transformer and YOLOv13, effectively capturing global contextual and local textural features for fine-grained recognition and analysis of tongue coating characteristics. Experimental results show that the enhanced model substantially outperforms the original YOLOv13 in fine-grained feature extraction and boundary localization, establishing a reliable foundation for the objectification, standardization, and intelligent advancement of tongue diagnosis in traditional Chinese medicine.

## 1. Introduction

Tongue inspection is a fundamental component of traditional Chinese medicine diagnosis, involving the analysis of tongue body and coating shape, color, and distribution to assess internal organ function and disease progression [1,2,3,4,5]. Among these features, tongue coating appearance is especially informative, as its variations reflect critical pathological states, including damp-cold, internal heat, deficiency-excess, and damp-turbidity [6,7]. However, traditional tongue diagnosis relies heavily on practitioner experience, and its outcomes are influenced by external factors, including lighting conditions, observation angles, and inter-practitioner variability. This reliance limits the objectivity and reproducibility of tongue diagnosis, posing a significant challenge to traditional Chinese medicine standardization and modernization. Therefore, developing an objective, standardized, and automated approach for tongue coating recognition and analysis is essential to advance the modernization and intelligent development of traditional Chinese medicine diagnostics.

Early studies mainly relied on handcrafted feature extraction methods—such as grayscale, texture, and color statistics—combined with shallow classifiers (e.g., support vector machines) for tongue image recognition [8,9]. Although these methods achieved acceptable accuracy under controlled conditions, they exhibited limited feature representation and poor robustness in complex backgrounds or variable lighting environments. In recent years, rapid advances in computer vision and deep learning have significantly promoted the automation and intelligent analysis of tongue diagnosis [10,11]. Researchers have progressively adopted convolutional neural network (CNN)-based object detection frameworks [12,13], whose efficient real-time detection capabilities have opened new avenues for advancing tongue image diagnosis.

However, the direct application of classical CNN models to tongue coating recognition remains challenging. First, the boundary between the tongue body and coating is often indistinct, with gradual color transitions, complicating precise segmentation and target region detection. Second, tongue coating images exhibit substantial intra-class variability, as thickness, color, moisture, and distribution patterns differ considerably across individuals. Furthermore, environmental factors during image acquisition—such as uneven lighting and varying capture angles—further increase data complexity and place greater demands on model robustness. Consequently, traditional CNN models often struggle to maintain high detection accuracy and generalization when handling such fine-grained, highly variable medical images.

To address challenges of indistinct boundaries between the tongue body and coating and subtle feature variations, the proposed YOLOv13-Swin model integrates a swin transformer into the YOLO detection framework, enhancing joint modeling of global and local features and improving fine-grained recognition of ambiguous tongue coating characteristics. The proposed approach aims to achieve high-precision detection and classification of tongue coating regions, promote the objectification and standardization of tongue diagnosis, and provide a reliable technical basis for developing intelligent auxiliary diagnostic systems in traditional Chinese medicine. The main contributions of this study are summarized as follows:

1. First application of YOLOv13 for tongue-coating diagnosis: This study pioneers the use of YOLOv13 in tongue image analysis, achieving efficient and objective detection of tongue coatings.
2. Hybrid YOLOv13-Swin architecture: By integrating a swin transformer into the YOLOv13 backbone, the network jointly models global and local features, enabling fine-grained recognition of subtle and ambiguous tongue-coating characteristics.
3. High-precision detection with clinical potential: Experimental results demonstrate superior accuracy in detecting and classifying tongue-coating regions, supporting the objectification and standardization of tongue diagnosis and providing a solid technical foundation for intelligent auxiliary diagnostic systems in traditional Chinese medicine.

The paper is organized as follows: Section 2 reviews related work; Section 3 details the proposed algorithm; Section 4 presents the experimental design and results; and Section 5 concludes the study and discusses future research directions.

## 2. Related Work

As a key element in the objectification and intelligent analysis of traditional Chinese medicine, tongue image analysis has progressively transitioned from traditional image processing methods to deep learning approaches, yielding a series of significant research outcomes [14,15]. This section systematically reviews and evaluates the current research landscape, emphasizing the technological evolution of tongue image analysis [16] and the adoption of object detection frameworks [17].

### 2.1. Advancements in Tongue Image Analysis Techniques

Early research on tongue image analysis mainly relied on traditional digital image processing techniques. These methods extracted handcrafted features—such as color, texture, and shape—from tongue images and integrated them with shallow machine learning models, including support vector machines (SVM) and K-nearest neighbors, for classification and recognition. Ding Jie et al. proposed a tongue image classification algorithm based on the doublet SVM to advance the objectification and intelligent analysis of traditional Chinese medicine tongue diagnosis [18]. The method improved classification accuracy by constructing sample pairs and incorporating histogram of oriented gradients (HOG) features. However, this approach exhibited several limitations. It primarily relied on grayscale and HOG features while neglecting multimodal information such as color, texture, and regional pathological variations of the tongue, thereby limiting its ability to comprehensively represent complex tongue characteristics. Zhang et al. proposed a tongue image analysis algorithm based on geometric features to quantitatively associate tongue shape with health status (healthy or diseased) [19]. However, this method relies solely on geometric information, leading to a limited feature dimensionality that constrains its ability to capture other diagnostically relevant characteristics in tongue images, such as color and texture. Zhang et al. developed an automated tongue image feature extraction and syndrome classification system designed to transform the subjective diagnostic process of traditional Chinese medicine tongue diagnosis into a quantifiable and computable model [20]. However, the feature extraction process still relied on handcrafted algorithms, which limited the model’s representational capacity and generalization performance.

### 2.2. Application of Machine Learning in Tongue Coating Detection

With the rapid advances in machine learning, CNN-based approaches have emerged as the dominant paradigm in tongue image analysis. CNNs can automatically learn hierarchical feature representations directly from raw images, substantially enhancing the accuracy of classification and segmentation. Hou et al. proposed an enhanced CaffeNet convolutional neural network for tongue color classification, integrating batch normalization layers and a dynamic learning rate strategy, and achieved 83% accuracy on a six-class task [21]. Zhou et al. proposed a tongue image-based constitution classification method that integrates gray-level co-occurrence matrix features, morphological characteristics, and a fine-tuned AlexNet, achieving 63% accuracy for three types of traditional Chinese medicine constitutions [22]. Liu et al. proposed a lightweight CNN–SVM hybrid model, integrating SAM-based segmentation and data augmentation techniques, and achieved 94% accuracy in tongue coating classification [23]. However, most studies remain focused on single tasks, such as tongue body segmentation or tongue color/coating classification, treating the tongue coating as a single, homogeneous category. These studies have yet to achieve detection and classification of specific tongue coating regions on the tongue body, which is essential for fine-grained syndrome differentiation in traditional Chinese medicine.

### 2.3. Application of YOLO-Based Object Detection in Medical Images

Object detection models, especially the YOLO series, have been widely applied in medical image analysis owing to their efficient end-to-end detection capabilities and superior real-time performance. YOLO formulates object detection as a regression problem, directly predicting bounding boxes and class probabilities within a single network. Its speed advantage makes it particularly suitable for rapid clinical diagnosis.

In tongue image analysis, researchers have increasingly explored the application of the YOLO framework. Li et al. proposed a multi-task tongue image recognition model based on YOLOv3, integrating transfer learning to simultaneously detect tongue color, coating color, fissures, and tooth marks [24]. Zhang et al. proposed a tongue coating color diagnostic system based on an enhanced YOLOv5s model [25]. The study aims to address diagnostic inconsistency in traditional Chinese medicine tongue diagnosis, which often results from reliance on practitioners’ subjective experience. However, most studies remain focused on single tasks, such as tongue body segmentation or tongue color/coating classification, treating the tongue coating as a single, homogeneous category. These studies have yet to achieve detection and classification of specific tongue coating regions on the tongue body, which is essential for fine-grained syndrome differentiation in traditional Chinese medicine.

## 3. Methodology

**Figure 1:**
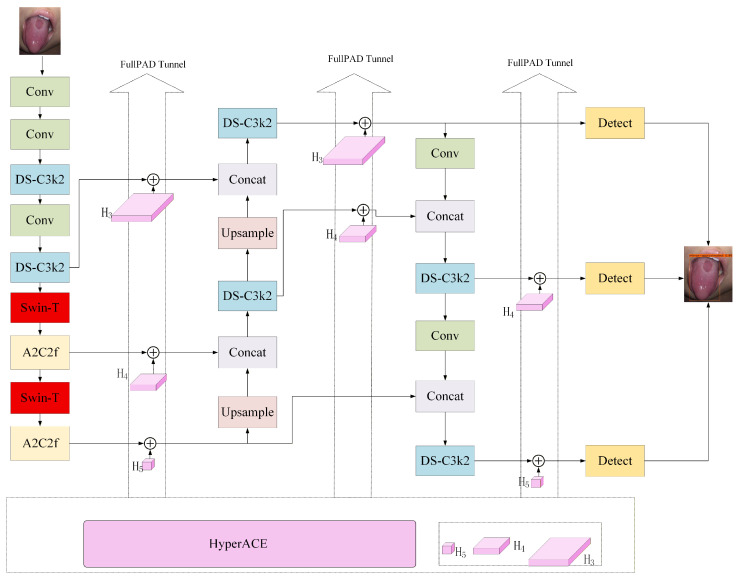
Intelligent tongue coating recognition method based on YOLOv13-Swin module.

As illustrated in Figure 1, an intelligent tongue coating recognition network based on YOLOv13 is proposed. The proposed method replaces the original depthwise separable convolution (DSConv) structure in the backbone with a swin transformer module, enhancing the ability to capture complex tongue coating textures and long-range dependencies. The overall architecture follows the standard three-stage YOLOv13 design, comprising the Backbone, Neck, and Head. The input tongue images are first processed by multiple convolutional layers and DS-C3k2 modules for initial feature extraction. At the intermediate-to-high semantic stages (feature scales H4 and H5), the original DSConv modules are replaced with swin transformer modules to model global context and enhance fine-grained tongue coating representations. The swin transformer–enhanced features are fused through the A2C2f module and fed into the YOLOv13 Neck, where multi-scale feature pyramids are constructed via upsampling and feature concatenation. Finally, the fused multi-scale features are delivered to the detection heads to enable accurate localization and classification of tongue coating regions across multiple scales.

### 3.1. Tongue Image Preprocessing

Tongue coating images were preprocessed following YOLOv13 procedures to ensure data standardization and training stability before network input. Raw tongue images were first resized and converted to the required format to meet network input specifications. Pixel values were normalized to the [0, 1] range to reduce the effects of illumination variations and imaging differences. During training, YOLOv13 data augmentation strategies—including random horizontal flipping, scaling, cropping, and slight rotation—were applied to increase sample diversity and improve model generalization. This preprocessing pipeline, consistent with YOLOv13, preserves semantic information while enhancing training stability and recognition performance.

### 3.2. Network Architecture Improvement

**Figure 2:**
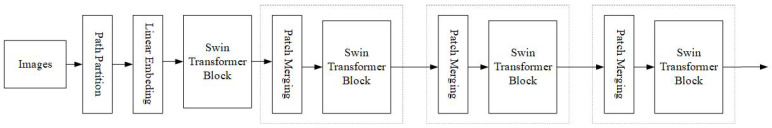
Swin Transformer Module.

In this study, key improvements were made to the YOLOv13 architecture by replacing the original DSConv modules with swin transformer modules, as shown in Figure 2, to enhance feature representation for tongue coating images. Swin transformer modules were introduced at critical feature extraction layers of YOLOv13 to capture global contextual information while preserving local feature extraction capabilities.

The swin transformer adaptively models local regions via a hierarchical shifted window attention mechanism. The shifting operation between windows enables effective cross-window interaction, yielding a global feature representation. Compared with traditional DSConv modules that extract features only within local receptive fields, Swin transformer captures subtle textures, color distributions, and edge information in tongue coating images more precisely, offering significant advantages for distinguishing tongue coating categories.

Furthermore, the swin transformer module offers scalability and hierarchical feature extraction, generating rich semantic features at multiple scales that align with YOLOv13’s multi-scale prediction architecture. During forward propagation, Swin-T features are fused with the A2C2f and HyperACE modules, enabling effective multi-scale integration and improving detection accuracy and robustness.

#### 3.2.1. Patch Partition and Linear Embedding

Given an input tongue image $I\in R^{H\times W\times 3}$, it is first divided into a set of non-overlapping patches of size P×P. Each patch is flattened and projected into a *C*-dimensional feature vector through a linear embedding layer:

(1) $$z_{0}=\left[x_{1}E;x_{2}E;\ldots ;x_{N}E\right]+E_{pos},\;x_{i}\in R^{P^{2}\times 3}$$

where $E\in R^{(P^{2}\cdot 3)\times C}$ denotes the learnable embedding matrix, $E_{pos}$ is the positional encoding, and $N=\frac{HW}{P^{2}}$ is the total number of patches.

#### 3.2.2. Window-Based Multi-Head Self-Attention (W-MSA)

To capture localized texture information such as coating smoothness and granularity, swin transformer computes self-attention within non-overlapping windows. For a window containing $M^{2}$ tokens, the attention operation is defined as [26]:

(2) $$\mathrm{Attention}\left(Q,K,V\right)=\mathrm{Softmax}\left(\frac{QK^{⊤}}{\sqrt{d}}+B\right)V$$

where $Q=XW_{Q}$, $K=XW_{K}$, and $V=XW_{V}$ are the query, key, and value matrices, *d* is the channel dimension of each head, and *B* denotes a learnable relative position bias added to preserve spatial relationships between local tongue regions.

#### 3.2.3. Shifted Window Mechanism

To enable cross-window communication and model long-range dependencies, the window partition is shifted by half the window size between consecutive layers. Let W−MSA and SW−MSA denote standard and shifted window attention operations, respectively. For each stage *l*, the output feature ${\hat{z}}_{l}$ and $z_{l+1}$ are computed as:

(3) $$\begin{array}{cc} {\hat{z}}_{l} & =\text{W-MSA}\left(\mathrm{LN}\left(z_{l-1}\right)\right)+z_{l-1} \end{array}$$

(4) $$\begin{array}{cc} z_{l} & =\mathrm{MLP}\left(\mathrm{LN}\left({\hat{z}}_{l}\right)\right)+{\hat{z}}_{l} \end{array}$$

(5) $$\begin{array}{cc} {\hat{z}}_{l+1} & =\text{SW-MSA}\left(\mathrm{LN}\left(z_{l}\right)\right)+z_{l} \end{array}$$

(6) $$\begin{array}{cc} z_{l+1} & =\mathrm{MLP}\left(\mathrm{LN}\left({\hat{z}}_{l+1}\right)\right)+{\hat{z}}_{l+1} \end{array}$$

This alternating application of window and shifted-window attention ensures efficient feature propagation between neighboring tongue regions, allowing the network to perceive coating continuity and boundary transitions effectively.

#### 3.2.4. Hierarchical Representation and Multi-Scale Feature Maps

The hierarchical design of swin transformer progressively merges neighboring patch tokens between stages through patch merging layers. After each merging operation, the spatial resolution is halved, and the feature dimension is doubled:

(7) $$z^{\left(s+1\right)}=\mathrm{PatchMerging}\left(z^{\left(s\right)}\right),\;z^{\left(s+1\right)}\in R^{\frac{H_{s}}{2}\times \frac{W_{s}}{2}\times 2C_{s}}$$

This yields a series of multi-scale feature maps $\left\{z^{\left(1\right)},z^{\left(2\right)},z^{\left(3\right)},z^{\left(4\right)}\right\}$, which encode fine-grained local textures and global semantic context simultaneously. For tongue coating analysis, low-level stages capture coating surface granularity and fissures, whereas higher stages encode global thickness distribution and chromatic variations.

### 3.3. YOLOv13 for Tongue Coating Detection and Diagnosis

As the latest advancement in the YOLO series, YOLOv13 introduces a hybrid detection architecture that combines anchor-based and anchor-free mechanisms with a task-aligned label assignment (TALA) strategy, establishing a robust foundation for precise tongue coating image analysis. In traditional Chinese medicine diagnosis, tongue coatings exhibit complex multi-scale patterns—ranging from thick, greasy coatings to thin-white or nearly mirror-like surfaces—that demand fine-grained detection and adaptive perception. By eliminating redundant encoding layers and enhancing feature interaction, YOLOv13 achieves high detection accuracy and interpretability suitable for medical imaging applications.

#### 3.3.1. Overall Architecture

The YOLOv13 detection pipeline consists of four main components:

(8) $$F_{\mathrm{out}}=\Phi_{\mathrm{head}}\left(\Phi_{\mathrm{neck}}\left(\Phi_{\mathrm{backbone}}\left(I\right)\right)\right)$$

where I denotes the input tongue coating image, $\Phi_{\mathrm{backbone}}$ extracts multi-level features, $\Phi_{\mathrm{neck}}$ fuses cross-scale contextual information, and $\Phi_{\mathrm{head}}$ performs classification and regression.

#### 3.3.2. Backbone: Hierarchical Tongue Feature Extraction

YOLOv13 adopts an improved CSPDarknet53 backbone, a variant of cross stage partial networks (CSPNet). Given the input image $I\in R^{H\times W\times 3}$, the hierarchical feature extraction process is expressed as:

(9) $$F^{\left(l\right)}=\sigma \left(\mathrm{ConvBNAct}\left(\mathrm{CSPBlock}\left(F^{\left(l-1\right)}\right)\right)\right)$$

where $F^{\left(l\right)}$ represents the feature map at layer *l*, and σ(·) denotes nonlinear activation. Shallow layers (l=1,2) capture fine-grained coating textures such as the granular surfaces of greasy-white tongues, while deeper layers (l>3) model global distribution patterns, such as the spread of yellow coatings toward the tongue edges. This hierarchical representation closely aligns with traditional Chinese medicine diagnostic logic, which considers both local microtexture and global morphology in coating analysis.

#### 3.3.3. Neck: Multi-Scale Fusion with SPD-Conv

To prevent the loss of diagnostically relevant fine details (e.g., cracks, tooth marks, or reflective patches), YOLOv13 integrates spatial pyramid dilation convolution (SPD-Conv) in the neck module. Given an intermediate feature map $F\in R^{H\times W\times C}$, SPD-Conv redistributes spatial information into the channel dimension:

(10) $$F^{\prime }=\mathrm{Reshape}\left({\mathrm{Conv}}_{k,d}\left(F\right)\right),\;F^{\prime }\in R^{\frac{H}{s}\times \frac{W}{s}\times \left(s^{2}\cdot C\right)}$$

where *k* is the convolution kernel size, *d* is the dilation rate, and *s* is the stride. This operation achieves spatial downsampling while retaining high-frequency coating information, effectively preserving microstructures crucial for distinguishing tongue coatings such as thin-white versus mirror-like types.

#### 3.3.4. Decoupled Detection Head

The detection head employs a decoupled architecture, independently processing the classification and regression branches to reduce task interference. For each feature level *i*, the detection prediction $Y_{i}$ is formulated as:

(11) $$Y_{i}=\left[C_{i}\left(F_{i}\right),\;R_{i}\left(F_{i}\right)\right]$$

where $C_{i}$ and $R_{i}$ denote the classification and regression subheads, respectively. The classification branch outputs the probability distribution over coating categories:

(12) $$p_{c}=\mathrm{Softmax}\left(W_{c}*F_{i}+b_{c}\right)$$

and the regression branch predicts bounding box coordinates $\hat{b}=\left(x,y,w,h\right)$ using:

(13) $$\hat{b}=W_{r}*F_{i}+b_{r}$$

In tongue coating analysis, the classification branch focuses on color, texture, and thickness identification, while the regression branch delineates coating boundaries with high precision. The decoupled design significantly improves the differentiation of visually similar coatings (e.g., greasy-white vs. thin-white) and enhances boundary localization accuracy.

#### 3.3.5. Loss Function with Task-Aligned Label Assignment (TALA)

YOLOv13 employs a task-aligned label assignment strategy to harmonize the optimization of classification and localization losses. For each predicted bounding box $b_{i}$, the total detection loss is defined as:

(14) $$L=\sum_{i=1}^{N}\left(\lambda_{cls}L_{cls}\left(p_{i},p_{i}^{*}\right)+\lambda_{reg}L_{reg}\left(b_{i},b_{i}^{*}\right)\right)$$

where $L_{cls}$ is the classification loss (typically focal loss), $L_{reg}$ is the regression loss (IoU-based), and $\lambda_{cls},\lambda_{reg}$ are balance coefficients. TALA dynamically matches ground-truth labels to predictions based on joint confidence and spatial alignment, ensuring that visually similar coatings are correctly assigned to their optimal anchor points.

#### 3.3.6. Adaptive Visual Perception for Traditional Chinese Medicine Tongue Diagnosis

By integrating hierarchical perception, fine-grained spatial encoding, and task-aligned optimization, YOLOv13 effectively adapts to the multi-scale and subtle visual patterns of tongue coatings. The complete detection process can be summarized as:

(15) $$\hat{Y}=\Psi_{\mathrm{YOLOv}13}\left(I;\Theta_{\mathrm{Swin}},\Theta_{\mathrm{YOLO}}\right)$$

where $\Theta_{\mathrm{Swin}}$ and $\Theta_{\mathrm{YOLO}}$ represent the parameters of the swin transformer feature extractor and the YOLOv13 detector, respectively. This synergistic design enhances the robustness, interpretability, and medical applicability of the model, providing an effective computational framework for intelligent traditional Chinese medicine tongue diagnosis.

## 4. Experiments

To comprehensively evaluate the proposed hybrid model for tongue coating recognition and classification, a series of experiments was conducted. Under a unified experimental setting, the hybrid model was compared with mainstream object detection algorithms—Faster R-CNN [27] and YOLOv13 [28]—to evaluate detection performance across tongue coating categories. To ensure robustness and generalization, a comprehensive dataset was constructed for training and validation under varying lighting conditions and shooting angles.

### 4.1. Experimental Setting

The tongue coating dataset used in this study was obtained from a publicly accessible online source. The dataset comprises 1407 tongue images spanning five clinically relevant categories: mirror-approximated, thin-white, white-greasy, yellow-greasy, and grey-black. All categories were manually annotated by experienced traditional Chinese medicine practitioners, ensuring label reliability and clinical relevance. The dataset was partitioned into training, validation, and test sets according to standard practice. The training set contains 937 images, the validation set 236 images, and the test set 234 images. This partitioning strategy facilitates effective model training and provides a reliable evaluation of model generalization in tongue coating detection and classification. All models were trained for 200 epochs with a batch size of 64 using NVIDIA RTX 3090Ti GPUs. Input images were resized to 640 × 640 pixels.

### 4.2. Experimental Comparison

**Figure 3:**
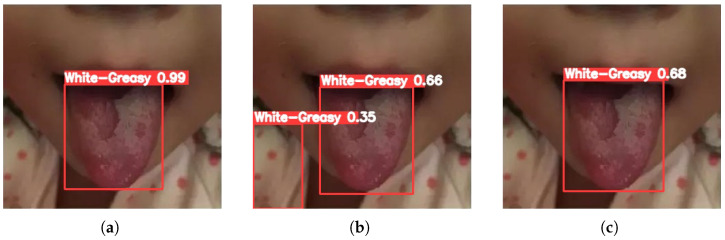
Detection results of white-greasy tongue coatings using different models: (**a**) Faster R-CNN, (**b**) YOLOv13, and (**c**) YOLOv13-Swin.

**Figure 4:**
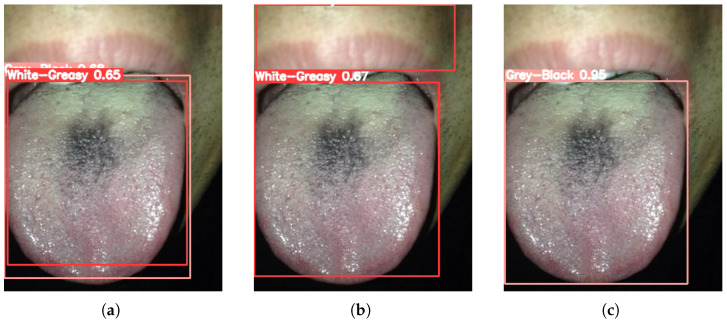
Detection results of grey-black tongue coatings using different models: (**a**) Faster R-CNN, (**b**) YOLOv13, and (**c**) YOLOv13-Swin.

**Figure 5:**
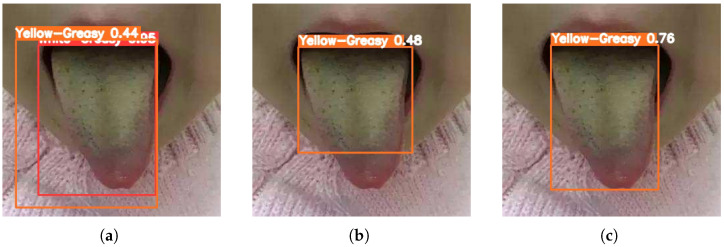
Detection results of yellow-greasy tongue coatings using different models: (**a**) Faster R-CNN, (**b**) YOLOv13, and (**c**) YOLOv13-Swin.

**Figure 6:**
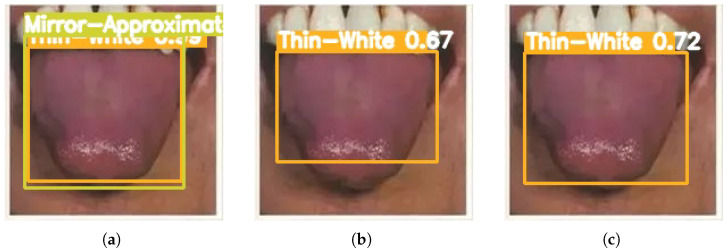
Detection results of thin-white tongue coatings using different models: (**a**) Faster R-CNN, (**b**) YOLOv13, and (**c**) YOLOv13-Swin.

**Figure 7:**
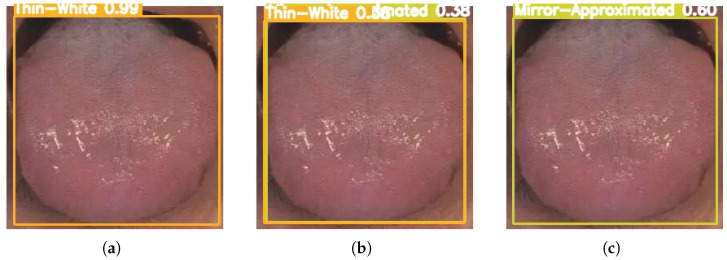
Detection results of mirror-approximated tongue coatings using different models: (**a**) Faster R-CNN, (**b**) YOLOv13, and (**c**) YOLOv13-Swin.

Figure 3, Figure 4, Figure 5, Figure 6 and Figure 7 compare the tongue coating detection results of the proposed YOLOv13-Swin, Faster R-CNN, and the original YOLOv13. For white-greasy (Figure 3), YOLOv13-Swin achieves more accurate localization with higher confidence scores than YOLOv13 and Faster R-CNN, effectively capturing tongue boundary features. For grey-black cases (Figure 4), the original YOLOv13 suffers from missed detections and low confidence scores, whereas YOLOv13-Swin consistently covers the full tongue region. In yellow-greasy samples (Figure 5), the swin transformer enhances color and texture feature extraction, leading to improved localization accuracy. For thin-white coatings (Figure 6), YOLOv13-Swin shows higher sensitivity to fine-grained distributions and produces clearer boundaries. In mirror-approximated cases (Figure 7), the global contextual modeling of the swin transformer facilitates reflective coating detection and reduces missed and false detections.

YOLOv13-Swin demonstrates higher detection accuracy and confidence than YOLOv13 and Faster R-CNN, particularly in fine-grained and low-contrast tongue regions. These results validate the effectiveness of replacing the DSConv module in YOLOv13 with the swin transformer.

Table 1 compares the proposed YOLOv13-Swin with the baseline YOLOv13 and the traditional object detector, Faster R-CNN. Although Faster R-CNN attains a moderate accuracy of 52.9% (${\mathrm{AP}}_{val}^{50:95}$), it demands substantial computation (215.59 GFLOPs, 41.32 M parameters) and has a high inference latency of 27.32 ms, rendering it unsuitable for real-time applications. In contrast, YOLOv13 greatly reduces computational complexity (6.2 GFLOPs) and parameters (2.45 M), with an inference time of 1.8 ms, but its accuracy is only 37.4% (${\mathrm{AP}}_{val}^{50:95}$).

YOLOv13-Swin replaces YOLOv13’s DSConv modules with the swin transformer, effectively enhancing feature extraction. Experiments demonstrate that YOLOv13-Swin achieves 63.1% ${\mathrm{AP}}_{val}^{50:95}$, 75% ${\mathrm{AP}}_{val}^{50}$, and 74.6% ${\mathrm{AP}}_{val}^{75}$ for tongue coating detection, with a latency of merely 0.7 ms. This significantly improves detection accuracy while maintaining exceptional real-time performance.

YOLOv13-Swin substantially improves tongue coating detection accuracy, retains a lightweight design, and enables fast inference. This demonstrates the benefits of integrating the swin transformer for enhanced local and global feature representation, validating the method’s superiority for this task.

### 4.3. Ablation Study

Table 2 reports the ablation results under different architectural configurations to evaluate the effect of replacing DSConv modules in YOLOv13 with the swin transformer. Specifically, YOLOv13-Swin (base) refers to the configuration in which a single DSConv module is replaced by the swin transformer, while the remaining modules retain the original DSConv design. YOLOv13-Swin denotes the configuration with all DSConv modules replaced by the swin transformer.

The baseline YOLOv13 exhibits limited tongue coating detection performance, achieving an ${\mathrm{AP}}_{val}^{50:95}$ of 37.4%, which suggests insufficient modeling of complex textures and global structures. Replacing a single DSConv module with the swin transformer (YOLOv13-Swin (base)) substantially improves performance, increasing ${\mathrm{AP}}_{val}^{50:95}$ to 62.5%, a gain of 25.1% over the baseline. This indicates that the swin transformer effectively captures global contextual and texture information in tongue coating images.

With full replacement of DSConv modules (YOLOv13-Swin), the model further improves to an ${\mathrm{AP}}_{val}^{50:95}$ of 63.1% while maintaining a lightweight parameter count of 2.52 M. Moreover, ${\mathrm{AP}}_{val}^{50}$ and ${\mathrm{AP}}_{val}^{75}$ reach 75.0% and 74.6%, respectively, indicating improved localization accuracy and detection robustness. The ablation results show that introducing the swin transformer into YOLOv13 yields consistent and interpretable performance gains, and deeper integration leads to further improvements without a significant increase in model complexity. This validates the effectiveness of the proposed YOLOv13-Swin architecture for tongue coating detection.

## 5. Conclusions

This study proposes a YOLOv13-Swin algorithm for fine-grained detection and classification of tongue coatings in traditional Chinese medicine. The proposed model integrates the strengths of the swin transformer and YOLOv13, enabling effective modeling of both local texture details and global structural features of tongue coatings. Experimental results demonstrate that the proposed model outperforms several mainstream deep learning methods in terms of average precision, while exhibiting improved stability, accuracy, and robustness across different tongue coating categories. These findings validate the potential of the proposed approach for application in automated and standardized traditional Chinese medicine diagnostic systems. Future work will focus on expanding dataset diversity, enhancing model interpretability, and incorporating multimodal diagnostic information (e.g., facial complexion and pulse signals) to further advance intelligent healthcare applications in traditional Chinese medicine.

Despite the encouraging performance achieved by the proposed YOLOv13-Swin model, several potential biases related to the dataset composition should be acknowledged. The tongue coating dataset used in this study was collected under relatively controlled conditions, and detailed demographic attributes, such as age and gender distributions, were not explicitly balanced during data acquisition. Consequently, the dataset may exhibit implicit biases toward specific age groups or gender characteristics, which could influence the learned feature representations. In addition, variations in imaging conditions, including illumination, camera devices, and acquisition environments, may introduce appearance discrepancies across tongue coating images. Although the proposed model demonstrates robustness on the validation set, its performance may be affected when applied to images captured under different lighting conditions or clinical settings. These factors may limit the generalization ability of the model when deployed in real-world clinical scenarios. Future work will focus on constructing larger and more diverse datasets with balanced demographic attributes and varied acquisition conditions, as well as incorporating domain adaptation and data augmentation strategies to further improve robustness and generalization performance.

## Figures and Tables

**Table 1:** Comparison of Faster R-CNN, YOLOv13, and YOLOv13-Swin.

Method	FLOPs (G)	Parameters (M)	${\mathrm{AP}}_{val}^{50:95}$	${\mathrm{AP}}_{val}^{50}$	${\mathrm{AP}}_{val}^{75}$	Latency (ms)
Faster R-CNN	215.59	41.32	52.9	59.6	52.9	27.32
YOLOv13	6.2	2.45	37.4	47.8	45.8	1.8
YOLOv13-Swin	-	2.52	63.1	75	74.6	0.7

**Table 2:** Impact of different modules on the performance of the YOLOv13 model.

Method	Parameters (M)	${\mathrm{AP}}_{val}^{50:95}$	${\mathrm{AP}}_{val}^{50}$	${\mathrm{AP}}_{val}^{75}$
YOLOv13	2.45	37.4	47.8	45.8
YOLOv13-Swin (base)	2.52	62.5	72.6	71.3
YOLOv13-Swin	2.52	63.1	75	74.6

## Data Availability

The tongue coating dataset used in this study is publicly available at https://download.csdn.net/download/qq_63961628/90880306 (20 December 2025). Researchers can freely access and download the dataset for academic purposes.

## Abbreviations

The following abbreviations are used in this manuscript:

CNN	Convolutional neural network
SVM	Support vector machines
HOG	Histogram of oriented gradients
W-MSA	Window-based multi-head self-attention
SPD-Conv	Spatial-to-depth convolution
TALA	Loss function with task-aligned label assignment
